# A screening for cerebral deoxygenation during VT ablations in patients with structural heart disease

**DOI:** 10.1007/s00392-024-02493-4

**Published:** 2024-07-16

**Authors:** Julian Müller, Lena Koch, Philipp Halbfass, Karin Nentwich, Artur Berkovitz, Sebastian Barth, Christian Wächter, Heiko Lehrmann, Thomas Deneke

**Affiliations:** 1 Department of Cardiology, Faculty of Medicine, University Heart Center Freiburg-Bad Krozingen, University of Freiburg, Freiburg im Breisgau, Germany; 2 Clinic for Interventional Electrophysiology, Heart Centre Bad Neustadt, Bad Neustadt a. d. Saale, Germany; 3 Department of Cardiology and Angiology, Philipps-University Marburg, Marburg, Germany; 4 Clinic for Electrophysiology, Klinikum Nuernberg, Campus South, University Hospital of the Paracelsus Medical University, Nuremberg, Germany

**Keywords:** VT ablation, Neuromonitoring, Near-infrared spectroscopy

## Abstract

**Background:**

Patients undergoing ventricular tachycardia (VT) ablation often present with structural heart disease (SHD) and reduced ejection fraction. Inducing VT by programmed electrical stimulation (PES) puts these patients at risk for hemodynamic instability and cerebral hypoperfusion.

**Objective:**

The present study screens for cerebral oxygen desaturation phases (ODPs) in patients undergoing VT ablation.

**Methods:**

Forty-seven patients (age 61 ± 14 years, 72% males) underwent ablation of sustained VT with simultaneous neuromonitoring using near-infrared spectroscopy (NIRS).

**Results:**

Analysis of NIRS signal identified ODPs in 29 patients (62%). ODPs were associated with a higher prevalence of ischemic heart disease (IHD) (45% vs. 11%, *p* = 0.024), previous VT episodes (*n* = 16 vs. 4, *p* = 0.018), and VTs inducible by PES (*n* = 2.4 vs. 1.2, *p* = 0.004). Patients with ODPs were more likely to be admitted to intensive care unit (ICU) (78% vs. 33%, *p* = 0.005) and had more in-hospital VT recurrences (24% vs. 0%, *p* = 0.034). No differences were observed in VT recurrence rates after hospital discharge (41.4% vs. 44.4%, *p* = 0.60) and left ventricular ejection fraction (34% vs. 38%, *p* = 0.567). IHD (OR: 32.837, *p* = 0.006), ICU admission (OR: 14.112, *p* = 0.013), and the number of VTs inducible at PES (OR: 2.705, *p* = 0.015) were independently associated with ODPs.

**Conclusions:**

This study registers episodes of cerebral hypoperfusion in 62% of patients undergoing VT ablation and identifies IHD and the number of VTs inducible at PES as possible risk factors for these episodes.

**Supplementary Information:**

The online version contains supplementary material available at 10.1007/s00392-024-02493-4.

## Introduction

Ventricular tachycardia (VT) is a common arrhythmia in various cardiomyopathies complicating the course of the disease. Although its occurrence can be idiopathic, VT is more commonly associated with structural heart disease (SHD), where it worsens prognosis and increases the risk of sudden cardiac death [1–3].

Catheter ablation is an effective treatment for VT [4, 5], with procedural success rates of 70% in experienced centers leading to fewer implantable cardioverter-defibrillator (ICD) therapies, fewer occurrences of electrical storm, and lower rates of cardiac hospitalization [6–8].

Patients undergoing VT ablation represent a high-risk population characterized by advanced stages of SHD, high rates of acute heart failure and mortality [7, 9] with higher complication rates than other electrophysiological procedures, and a procedure-related mortality rate of 0.5% [10]. The safety of the procedure is, therefore, an important prerequisite for more widespread use of VT ablation.

Various efforts have been made to identify and minimize the risks inherent to VT ablation [11–13]. One major concern constitutes the hemodynamic instability that is provoked by the systematic induction of VT during the procedure. Although several new approaches to identify VT circuits are recently published, activation mapping during running VT remains a common approach in hemodynamically stable patients [14]. As most patients with ventricular tachycardia suffer from reduced cardiac contractility and take medication interfering with vascular reactivity, rapid ventricular pacing and induction of VTs might easily deteriorate this fragile system. Neuromonitoring during ICD testing corroborates that cardiovascular compensation mechanisms are often not competent enough to avert cerebral hypoperfusion in these settings [15–17].

To prevent intraoperative phases of cerebral oxygen desaturation and neurocognitive impairment following medical procedures, neuromonitoring is already an integral part of many surgical procedures. A few studies suggest that episodes of cerebral oxygen desaturation occur during VT ablation [18, 19]. To this point, the frequency and duration of these episodes remain unknown. Using NIRS neuromonitoring, the present study aims to identify episodes of cerebral oxygen desaturation during VT ablation, to characterize these episodes, and to identify patients at risk for cerebral hypoperfusion.

## Methods

### Study population

Patients presenting with sustained monomorphic VTs for ablation were included in this retrospective single-center study. Patient recruitment took place between June 2018 and May 2021 according to the consecutive presentation of patients at the Heart Center Bad Neustadt, Germany. Two out of 49 patients were excluded from further analysis due to the poor quality of NIRS data. VT was documented by either ICD storage, external defibrillator monitoring, or 12-lead ECG. SHD was diagnosed according to the respective European guidelines [20, 21]. Patients were followed for a mean of 632 ± 73 days. Written informed consent was obtained from all patients, and the study was approved by the institutional review board of the Heart Center Bad Neustadt, Germany.

### Electrophysiological study

Patients underwent VT ablation under conscious sedation achieved by continuous use of propofol and morphine derivatives. If necessary, transvenous or arterial catheter ablation was complemented with an epicardial ablation approach using a percutaneous subxiphoid approach, as previously described [22].

Programmed electrical stimulation (PES) was performed at the beginning of the procedure from at least two ventricular sites using up to four extrastimuli with two different cycle lengths (CL). VTs induced by PES were compared with the acquired VT documents for QRS morphology and CL and were classified as clinical VTs if both QRS morphology and CL matched. A hybrid mapping technique was administered in which all late potentials and local abnormal activity were first mapped to the anatomical map, and substrate mapping was performed. In a second step, if hemodynamically feasible, VT was induced, and activation mapping was performed with special attention to the previously marked suspicious areas. Otherwise, substrate mapping was performed in sinus rhythm, and all areas with low voltage (defined as voltage < 1.5 mV and > 0.5 mV), local abnormal electrical activity, or late potentials within the scare were ablated. This two-step procedure was performed to keep the duration of ongoing VTs as short as possible. For substrate mapping, 3D voltage maps were acquired using high-density 3D electroanatomic mapping (EAM) systems (Carto 3, BiosenseWebster; Ensite Precision, Abbott; or Rhythmia, Boston Scientific).

Ablation was performed with radiofrequency energy of 45 to 50 W and to a single-point target ablation index of 700 to 1000 using Carto. Using an Ensite or Rhythmia system energy was delivered to an impedance drop of at least 10% from baseline. Ablation of one site was stopped if local electrograms were eliminated. *Partial procedural success* was defined as the non-inducibility of the clinical VT combined with sustained inducibility of non-clinical VTs. *Full procedural success* was defined as the non-inducibility of all VTs, clinical and non-clinical.

### Near-infrared spectroscopy

NIRS uses the different chromophoric properties of oxygenated and deoxygenated hemoglobin to derive the concentrations of these molecules in superficial cortical tissue. Light of different wavelengths in the near-infrared spectrum is emitted, and the ratio of light emitted and refracted by the tissue is measured. This ratio can be converted into the concentrations of oxygenated and deoxygenated hemoglobin. The tissue oxygenation index (TOI) is determined by the percentage of oxygenated hemoglobin compared to total hemoglobin. Common TOI values range around 75%.

**Fig. 1:**
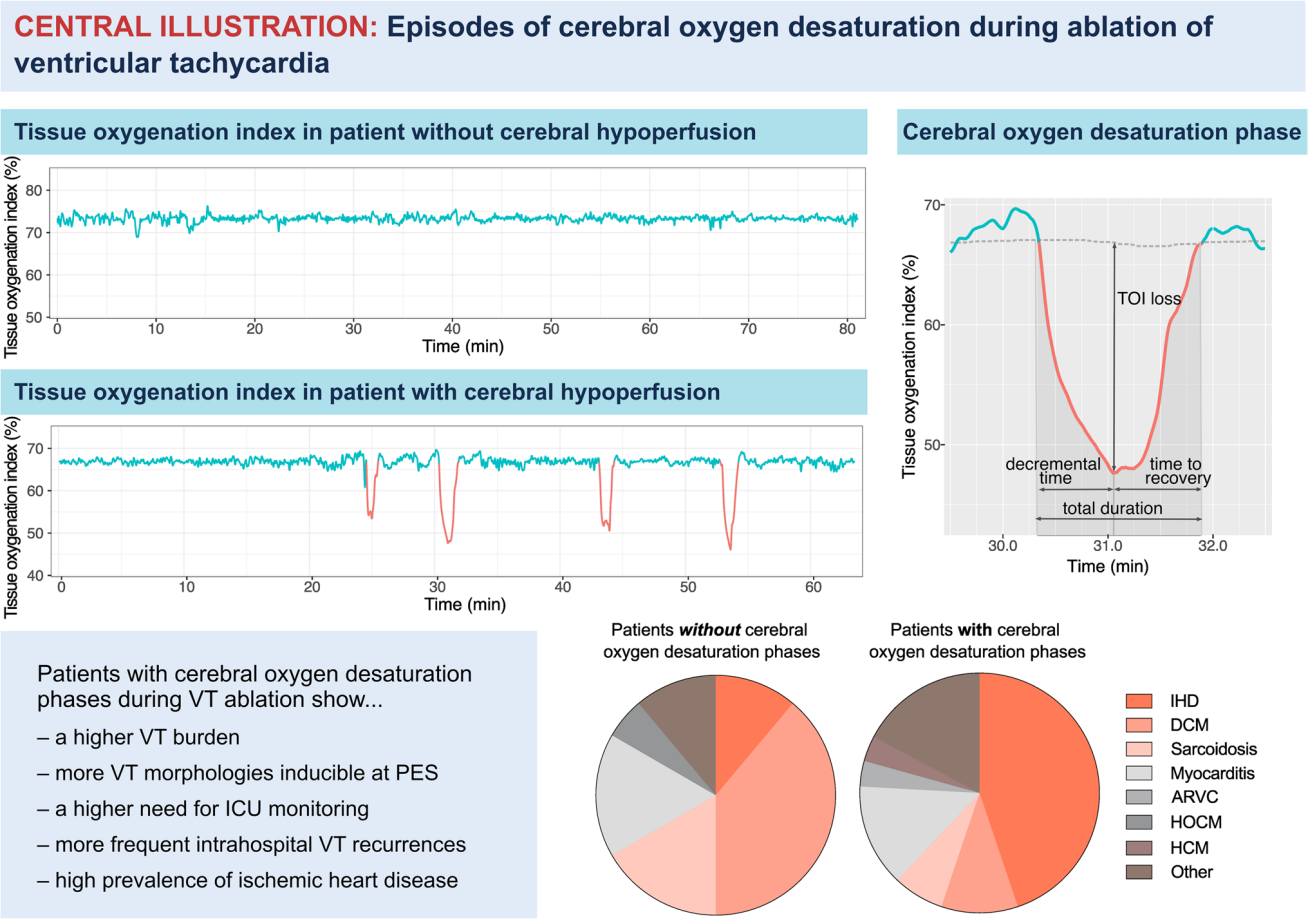
Episodes of cerebral oxygen desaturation during ablation of ventricular tachycardia. ARVC, arrhythmic right ventricular disease; DCM, dilated cardiomyopathy; HCM, hypertrophic cardiomyopathy; HOCM, hypertrophic obstructive cardiomyopathy; IHD, ischemic heart disease, ODP, oxygen desaturation phase; PES, programmed electrical stimulation; TOI, tissue oxygenation index; VT, ventricular tachycardia

Regional oxygen saturation was measured continuously during VT ablation using the NIRO-200NX device (Hamamatsu, Hamamatsu; Japan). The method has been validated for the NIRO-200NX device correlating TOI with neurological deficits and jugular bulb oxygen saturation [23, 24]. Two adhesive optodes were placed firmly on the forehead over the right and left frontal lobes with an interoptode distance of 4–6 cm. TOI data were further processed in R v4.3.1 (R-project.org). In five patients, the signal from one channel was of low quality, and therefore, this channel was excluded. For all other patients, the signal was averaged between the two channels. Rolling average correction was performed using a running median from a time window of 200 s (Fig. S1). Smoothing was performed over a time window of 5 s. To identify episodes of reduced oxygenated hemoglobin levels, TOI signal was analyzed for local minima defined as a drop in TOI of ≥ 6% below the rolling average for at least 15 s. These time periods were termed oxygen desaturation phases (ODPs) and were analyzed for (1) the *decremental time*, defined as the time to the trough of the desaturation phase, (2) the *time to recovery*, defined as the time until TOI signal recovered to baseline, (3) the *total duration* of the phase, and (4) *TOI loss*, defined as the drop in TOI signal to the trough of an ODP (Fig. 1). When an ODP was registered, VTs were terminated immediately by overdrive pacing or cardioversion/defibrillation.

### Periprocedural management

All patients underwent a comprehensive clinical assessment. Medical records were reviewed for past medical history and diagnosis of SHD. VT burden was defined as the number of previous VT episodes documented by ECG or device storage. Left ventricular ejection fraction (LVEF) was calculated according to the Simpson method from transthoracic echocardiography performed before discharge or in a few cases in the ICU or during admission. After VT ablation, patients were monitored for adverse events and in-hospital VT recurrence until discharge. If epicardial ablation was performed, an epicardial drainage was placed for at least 12 h following the procedure in case of pericardial bleeding.

### Statistical methods

Continuous variables were summarized as mean ± standard deviation (SD) and analyzed using either the Mann–Whitney U test or Student’s *t*-test depending on the assumption of normal distribution as assessed by the Shapiro-Wilk test. Categorical data were presented as quantity with percentages in brackets and analyzed using Fisher’s test. Simple linear regression was calculated to assess the influence of LVEF on TOI loss. Multivariate logistic regression was performed to identify predictors of ODPs from all variables with *p* < 0.100 in univariate statistical testing. Variables were excluded using stepwise backward selection until all variables met the prespecified criterion of *p* < 0.200. Statistical tests were performed with a two-tailed alternative hypothesis and comparisons with *p* values to fall below *p* < 0.05 were considered as significant. Data analysis and visualization were performed using R v4.3.1 (R-project.org) and GraphPad Prism v10.0.3.

## Results

### Study population

A total of 47 patients presenting with sustained monomorphic VT underwent VT ablation with intraprocedural continuous NIRS registration at our tertiary center between June 2018 and May 2021. Patient characteristics are shown in Table 1. Patients were 61.3 ± 14.4 years old, and 34 (72.3%) patients were male. IHD (31.9%) and DCM (22.2%) were the most common cardiomyopathies. Medications for heart failure, arrhythmia, and coronary artery disease (CAD) were used as shown in Table 1. The mean LVEF was 35.1 ± 14.4%. Twenty-one patients (46.7%) suffered from acute heart failure during VT and hospital admission. Thirty patients (65.2%) had a transvenous ICD system, and 8 (17.4%) had a cardiac resynchronization therapy defibrillator (CRT-D).

**Table 1:** Demographic characteristics

	Overall (*n* = 47)	Patients without ODPs (*n* = 18)	Patients with ODPs (*n* = 29)	*p* value
Age (yrs)	61.3 ± 14.4	59.1 ± 12.2	62.7 ± 15.6	0.231
Sex (male)	34 (72.3)	11 (61.1)	23 (79.3)	0.199
CVRF				
Arterial hypertension	31 (67.4)	12 (66.7)	19 (67.9)	1.000
Diabetes mellitus	14 (30.4)	5 (27.8)	9 (32.1)	1.000
Hyperlipidemia	28 (60.9)	8 (44.4)	20 (71.4)	0.121
Smoking	16 (34.8)	5 (27.8)	11 (39.3)	0.533
Cardiac family history	6 (13.0)	2 (11.1)	4 (14.3)	1.00
Comorbidities				
Atrial fibrillation	20 (43.5)	7 (38.9)	13 (46.4)	0.763
Prior stroke	3 (6.5)	1 (5.6)	2 (7.1)	1.000
Coronary artery disease	21 (44.7)	3 (16.7)	18 (58.6)	0.006*
Chronic kidney disease	19 (41.3)	8 (44.4)	11 (39.3)	0.767
COPD	1 (2.2)	1 (5.6)	0 (0)	0.400
Structural heart disease				
DCM	10 (22.2)	7 (41.2)	3 (10.7)	0.027*
IHD	15 (31.9)	2 (11.1)	13 (44.8)	0.024*
Sarcoidosis	5 (10.9)	3 (16.7)	2 (7.1)	0.365
Myocarditis	7 (15.2)	3 (16.7)	4 (14.3)	1.000
Other	10 (20.8)	3 (16.7)	7 (24.1)	0.719
Prior medication				
Beta-blocker	40 (87.0)	16 (88.9)	24 (85.7)	1.00
Amiodarone	21 (45.7)	5 (27.8)	16 (57.1)	0.072
ACE inhibitor	13 (28.3)	5 (27.8)	8 (28.6)	1.000
ARB	26 (56.5)	9 (50)	17 (60.7)	0.550
MRA	32 (69.6)	11 (61.1)	21 (72.4)	0.345
ARNI	22 (47.8)	7 (38.9)	15 (53.6)	0.378
Diuretic agent	26 (56.5)	11 (61.1)	15 (53.6)	0.763
Statins	21 (45.7)	5 (27.8)	16 (57.1)	0.072
Type of ICD				
ICD	30 (65.2)	10 (55.6)	20 (71.4)	0.347
CRT-D	8 (17.4)	4 (22.2)	4 (14.3)	0.693
s-ICD	1 (2.2)	0 (0)	1 (3.6)	1.000
Life-vest	1 (2.2)	1 (5.6)	0 (0)	0.391
LVEF (%)	35.1 ± 14.4	37.6 ± 18.5	33.5 ± 11.4	0.567
Acute heart failure	21 (46.7)	6 (33.3)	15 (55.6)	0.223
Cardiogenic shock	1 (2.2)	1 (5.6)	0 (0)	0.400

Values are given as *n* (%) or mean ± standard deviation. Significant *p* values are displayed in bold print and marked with an asterisk

*Abbreviations*: *ACE*, angiotensin-converting enzyme; *ARB*, angiotensin receptor blocker; *ARNI*, angiotensin receptor-neprilysin inhibitor; *ARVC*, arrhythmic right ventricular disease; *COPD*, chronic obstructive pulmonary disease; *CRT-D*, cardiac resynchronization therapy-defibrillator; *CVRF*, cardiovascular risk factors; *DCM*, dilated cardiomyopathy; *HCM*, hypertrophic cardiomyopathy; *HOCM*, hypertrophic obstructive cardiomyopathy; *IHD*, ischemic heart disease; *LVEF*, left ventricular ejection fraction; *MRA*, mineralocorticoid receptor antagonist; *ODP*, oxygen desaturation phase; *(s-)ICD*, (subcutaneous) implantable cardioverter-defibrillator; *VT*, ventricular tachycardia

### Procedural characteristics

The procedural characteristics are shown in Table 2. Twenty-four patients (52%) had previously undergone VT ablation. During the initial PES, 1.9 ± 1.5 VTs per patient were inducible. However, in 18% of patients, no VT was induced with PES. Twelve cases (26%) underwent a combined endo/epicardial or epicardial-only approach, and in 28%, a transseptal approach to the left ventricle was chosen. To restore hemodynamic stability, 3 patients received catecholamines during the procedure, and 27 patients (60%) were admitted to the ICU before or after VT ablation. PES was repeated at the end of the procedure in all but one procedure, showing partial ablation success present in 96% of cases. Complete ablation success was achieved in 81% of cases. After the procedure, 7 patients (15%) had spontaneous in-hospital recurrence of VT, of whom 6 (14%) underwent reablation during the same hospital stay.

**Table 2:** Procedural characteristics

	Overall (*n* = 47)	Patients without ODPs (*n* = 18)	Patients with ODPs (*n* = 29)	*p* value
Prior VT ablation	24 (52.2)	7 (38.9)	17 (60.7)	0.227
Clinical VT CL (ms)	387.3 ± 91.9	393.6 ± 95	382.2 ± 91.5	0.848
VT burden	11.7 ± 20.7	4.3 ± 4.5	16.4 ± 25.4	0.018*
Procedural duration (min)	167.9 ± 78.0	153.2 ± 55.0	176.4 ± 58.8	0.193
Fluoroscopy duration (min)	15.1 ± 10.8	15.3 ± 12.4	15.0 ± 9.9	0.979
Ablation time (min)	37.4 ± 21.3	32.5 ± 22.2	40.3 ± 20.6	0.231
Endocardial ablation	43 (91.5)	15 (83.3)	28 (96.6)	0.15
Epicardial ablation	12 (25.5)	5 (27.8)	7 (24.1)	1.000
VTs inducible with initial PES	1.9 ± 1.5	1.2 ± 1.2	2.4 ± 1.4	0.004*
Noninducibility with initial PES	8 (17.8)	1 (5.9)	7 (24.1)	0.124
Transseptal puncture	13 (27.7)	5 (27.8)	8 (27.6)	1.000
Intraprocedural hemodynamic instability	10 (21.7)	3 (16.7)	7 (24.1)	0.717
Intraprocedural catecholamines	3 (6.7)	2 (11.1)	1 (3.7)	0.555
PES after ablation	46 (97.9)	18 (100)	28 (96.6)	1.000
Partial ablation success	45 (95.7)	18 (100)	27 (93.1)	1.000
Full ablation success	38 (80.9)	17 (94.4)	21 (72.4)	0.124
ICU monitoring	27 (60.0)	6 (33.3)	21 (77.8)	0.005*
Intrahospital VT recurrence	7 (14.9)	0 (0)	7 (24.1)	0.034*
Re-ablation during the same admission	6 (14.3)	0 (0)	6 (20.7)	0.066
MACE	21 (44.7)	8 (44.4)	13(44.8)	0.760
Death	1 (2.1)	0 (0)	1 (3.4)	1.000
Out of hospital VT recurrence	20 (42.6)	8 (44.4)	12 (41.4)	1.000

Values are given as *n* (%) or mean ± standard deviation. Significant *p* values are displayed in bold print and marked with an asterisk

*Abbreviations*: *CL*, cycle length; *ICU*, intensive care unit; *MACE*, major adverse cardiovascular events; *ODP*, oxygen desaturation phase; *PES*, programmed electrical stimulation; *VT*, ventricular tachycardia

### Cerebral oxygen desaturation phases

**Fig. 2:**
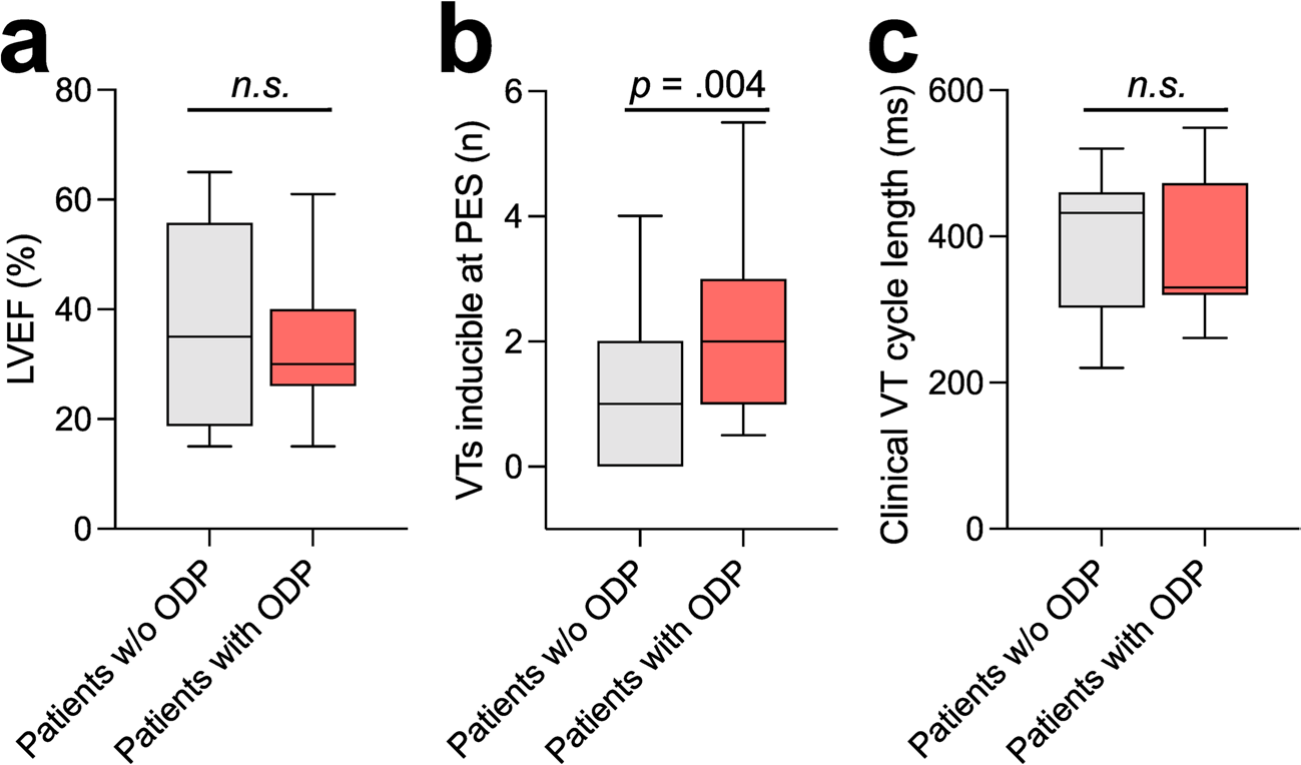
Comparison of **a** LVEF, **b** number of VTs inducible at initial PES, and **c** clinical VT cycle length between patient with and without ODPs. Whiskers display 5%– and 95%-percentiles. LVEF, left ventricular ejection fraction; ODP, oxygen desaturation phase; PES, programmed electrical stimulation; VT, ventricular tachycardia; w/o, without

A total of 90 ODPs were recorded across all VT ablation procedures. ODP characteristics are shown in Table 3. Importantly, these phases were not evenly distributed across patients: 18 patients (38.3%) did not experience a desaturation phase. In the remaining 29 patients (61.7%), 3.1 ± 1.6 ODPs were observed per procedure (Fig. 1). ODPs lasted 54.5 ± 30.3 s, and TOI loss was − 10.2 ± 5.5% (distribution of TOI loss is shown in Figure S2). The TOI signal decreased for 29.4 ± 21.0 s to reach the trough and took 25.0 ± 17.0 s to recover. A decrease in TOI greater than 10% occurred in 14 patients (Fig. 2).

**Table 3:** Characteristics of oxygen desaturation phases

Patients with ODPs	29 (61.7)
ODPs per patient	3.1 ± 1.6
TOI loss (%)	-10.2 ± 5.5
Decremental time (sec)	29.4 ± 21.0
Time to recovery (sec)	25.0 ± 17.0
Total duration (sec)	54.5 ± 30.3
Patients with TOI loss > 10%	14 (29.8)

Values are given as *n* (%) or mean ± standard deviation

*Abbreviations*: *ODP*, oxygen desaturation phase; *TOI*, tissue oxygenation index

### Clinical characteristics associated with oxygen desaturation phases

Patients with ODPs during VT ablation did not differ from patients without ODPs in terms of age (62.7 ± 15.6 years vs. 59.1 ± 12.2 years, *p* = 0.231), sex (79.3% vs. 61.1%, *p* = 0.199), or distribution of cardiovascular risk factors (Table 1). However, CAD had a significantly higher prevalence in patients with ODPs (58.6% vs. 16.7%, *p* = 0.006). Correspondingly, IHD was also significantly more common in patients with ODPs (44.8% vs. 11.1%, *p* = 0.024), whereas DCM was more common in patients without ODPs (10.7% vs. 41.2%, *p* = 0.027) (Fig. 1).

**Fig. 3:**
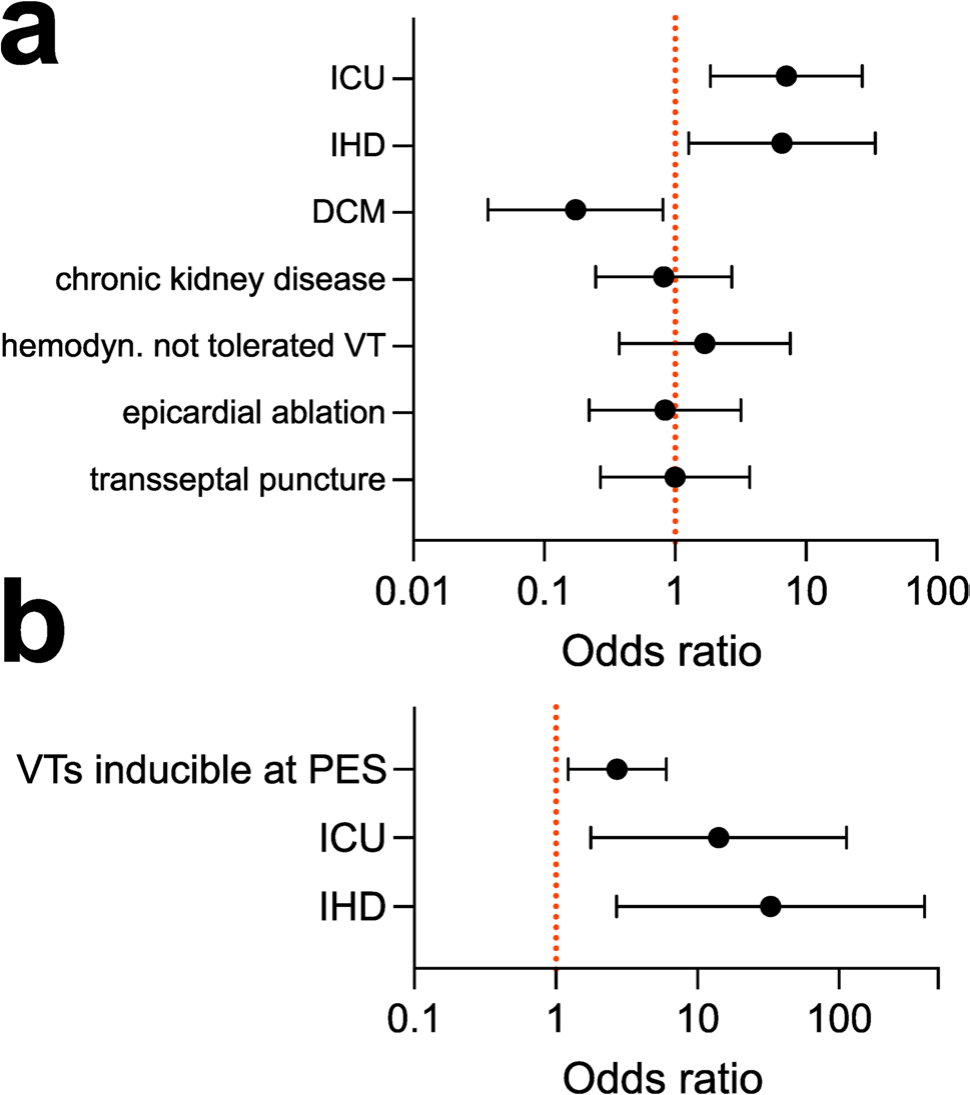
**a** Odds ratios to encounter ODPs during VT ablation from univariate statistics for selected variables. **b** Odds ratios of independent predictors of ODPs as computed from multivariate logistic regression. DCM, dilated cardiomyopathy; ICU, intensive care unit; IHD, ischemic heart disease; ODP, oxygen desaturation phase; PES, programmed electrical stimulation; VT, ventricular tachycardia

The groups did not differ in basic procedural characteristics such as ablation time (40.3 min vs. 32.5 min, *p* = 0.231), epicardial ablation (24.1% vs. 27.8%, *p* = 1.000), or transseptal access to the LV (27.6% vs. 27.8%, *p* = 1.000) (Fig. 3). There was also no difference in VT CL (382 ms vs. 393 ms, *p* = 0.848) (Fig. 2). Strikingly, however, patients with ODPs had a significantly higher VT burden with 16.4 ± 25.4 VTs documented before ablation as compared to 4.3 ± 4.5 in patients without ODPs *(p* = 0.018). Correspondingly, patients with ODPs had a higher number of VTs inducible at initial PES (*n* = 2.4 vs. 1.2, *p* = 0.004).

Intraprocedural hemodynamic instability, defined as sustained hypotension (i.e., systolic blood pressure < 80 mmHg), occurred in 22% of patients but was not more common in patients with ODPs (24.1% vs. 16.7%, *p* = 0.717). There was also no difference between the study populations with respect to LVEF (33.5% vs. 37.6%, *p* = 0.567) (Fig. 2), acute heart failure (55.6% vs. 33.3%, *p* = 0.223), or cardiogenic shock, which occurred in only one patient who did not experience ODPs (0% vs. 5.6%, *p* = 0.400) nor did LVEF predict the magnitude of TOI loss (*R*^2^ = 0.003, *F*(1,27) = 0.089, *p* = 0.767) (Figure S3). Interestingly, there was also no association between LVEF and intraprocedural hemodynamic instability (34.5% vs. 35.4%, *p* = 0.871).

**Fig. 4:**
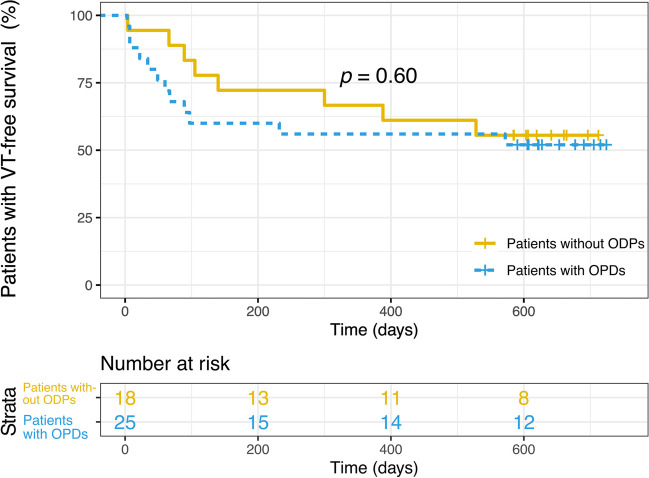
Kaplan–Meier curves illustrating VT recurrences stratified according to patients with and without ODPs. ODP, oxygen desaturation phase; VT, ventricular tachycardia

Despite a numerically lower full success rate in patients with ODPs, there was no significant difference in acute procedural success between patients with and without ODPs (partial success: 93.1% vs. 100%, *p* = 1.000, full success: 72.4% vs. 94.4%, *p* = 0.124). After ablation, patients with ODPs were more likely to be admitted to the ICU (77.8% vs. 33.3%, *p* = 0.005) and had more in-hospital VT recurrences (24.1% vs. 0.0%, *p* = 0.034). Patients were followed for 631.5 ± 72.8 days. One patient with ODPs died and 20 patients experienced a VT recurrence during the follow-up period. Out-of-hospital VT recurrence did not differ between groups (41.4% vs. 44.4%, log-rank *p* = 0.600) (Fig. 4). Multivariate logistic regression identified IHD (OR: 32.837, *p* = 0.006), the number of VTs inducible at initial PES (OR: 2.705, p = 0.015), and the need for ICU monitoring (OR: 14.112, *p* = 0.013) as independent predictors of ODPs during VT ablation.

## Discussion

The present study quantifies the prevalence of cerebral ODPs during VT ablation using NIRS neuromonitoring. Most importantly, ODPs occur in two-thirds of patients undergoing VT ablation. IHD, ICU admission, and the number of VTs induced during initial PES can predict intraprocedural ODPs. Interestingly, LVEF is not predictive of the occurrence or severity of ODPs. In conclusion, periods of cerebral hypoperfusion are a common event during VT ablation and are not adequately prevented by a hemodynamic management guided by standard blood pressure monitoring.

The hemodynamic management during VT ablations can be challenging due to underlying SHD and poor LV performance. Nevertheless, mean arterial pressure (MAP) remains the only parameter routinely used to guide hemodynamic management during VT ablation. Cerebral oxygenation is unreliably predicted by MAP as it is influenced by local factors such as carotid stenosis and cerebral autoregulation [25, 26]. Additional NIRS monitoring might therefore be a useful tool to enhance procedural safety in those patients.

We have identified phases of cerebral oxygen desaturation in two-thirds of patients undergoing VT ablation. EEG changes reflecting altered brain function typically follow cerebral hypoperfusion with a short delay—for the onset of ventricular fibrillation, this delay has been reported to be approximately 12 s [15]. Consequently, for a short period of time, brain function remains unaffected by cerebral deoxygenation, and rapid action, guided by NIRS monitoring, has the potential to prevent neuronal damage.

Studies evaluating the influence of ODPs on neurocognitive dysfunction have been mainly conducted in cardiac surgery. While most of these studies have found an association between ODPs and worse short- and long-term neurocognitive outcomes, the evidence on the matter remains inconsistent [27, 28]. Continuous NIRS monitoring administered during surgery has the potential to reduce prolonged perfusion deficits, postoperative cognitive dysfunction, and morbidity and mortality [27, 29]. There is also evidence that ODPs during VT ablation can be reduced using percutaneous left ventricular assist device (pLVAD) [19]. However, evidence on the prevalence, severity, and duration of ODPs during VT ablation is scarce, and there is no evidence on the neurocognitive effect of ODPs during VT ablation. Recent studies in patients with cardiogenic shock have highlighted the potential risks of mechanical circulatory support [30, 31]. A retrospective analysis of the use of pLVADs in VT ablation also failed to show a benefit from the device used for the composite endpoint of death, permanent pLVAD implantation, or heart transplantation [32]. Therefore, the use of these devices should be evaluated cautiously for a potential benefit in VT ablation.

Interestingly, patients with ODPs have a higher VT burden, more VTs inducible at initial PES, numerically lower acute ablation success, and more in-hospital VT recurrences. This suggests that ODPs occur in patients with higher arrhythmic activity. In line with this, the proportion of patients taking amiodarone was numerically higher in patients with ODPs. Notably, ODPs were also associated with in-hospital VT recurrences This could possibly be due to a less aggressive ablation strategy in patients who seemed hemodynamically compromised. Another possible reason could be a more complex substrate, resulting in both lower short-term success rates and the need for longer VT run times to map the VT. However, as we did not time-track VT induction during the procedure, we cannot substantiate this theory.

Contrary to our expectations, patients with a higher VT burden appear to be less adapted to the hemodynamic challenges of VT. A possible explanation is provided by evidence from animal studies suggesting a VT-induced cerebrovascular dysregulation that persists for hours after a VT episode [33]. If VT disrupts cerebrovascular autoregulation, frequent VT recurrences would not promote but rather impede appropriate cerebrovascular compensatory mechanisms.

The occurrence of ODPs was also associated with a higher number of ICU admissions. In retrospect, it is difficult to determine the reason for this observation as intraprocedural hemodynamic instability or the use of catecholamines did not account for this observation. Possible reasons could be that the procedures in which ODPs occurred were more complex, or that patients with ODPs were generally more severely ill, leading the clinician to choose post-procedural monitoring in the ICU. It is also possible that patients with ODPs had a worse cognitive outcome in the recovery room, leading to subsequent monitoring in the ICU. However, as no neurocognitive testing was performed, this reasoning can only be speculative, and further evidence is needed to determine if ODPs have short- and long-term effects on neurocognitive function and whether measures should be taken to prevent these episodes. Interestingly, neuromonitoring may lead to a reduction in ICU admissions in cardiac surgery [29]. Further research should therefore investigate whether neuromonitoring during VT ablation may have similar beneficial effects.

### Ischemic heart disease as risk factor for cerebral oxygen desaturation phases

IHD was found to be a strong predictor for the occurrence of ODPs, whereas the prevalence of DCM was negatively associated with ODPs. VT substrate in IHD and DCM differs dramatically: Ischemic scar tissue typically exhibits a confluent late gadolinium enhancement (LGE) involving the subendocardial layer, in contrast to midwall or subepicardial LGE in DCM [34]. Interestingly, these differences correlate with a higher VT recurrence after VT ablation in patients with DCM as compared to IHD [35, 36]. Although VT ablation in IHD has higher success rates and is often less complex, IHD appears to be closely associated with intraprocedural cerebral hypoperfusion.

We hypothesize that the predisposition for ODPs is determined by a combination of low cardiac output, volume status, and local factors regulating cerebral perfusion. In patients with IHD, the underlying coronary atherosclerosis is often part of a more generalized atherosclerotic disease. Thus, IHD is closely associated with carotid stenosis, cerebrovascular disease, and an increased risk of stroke and cognitive impairment [37, 38]. It is possible that the observed association between IHD and ODPs is due to comorbid cerebrovascular atherosclerosis, which in turn limits cerebral autoregulation. This term refers to the ability of the intracranial vasculature to maintain a steady cerebral perfusion independent of blood pressure fluctuations. Caldas et al. found that cerebral autoregulation in patients with IHD is severely impaired, as it responds much more slowly to counterbalance fluctuations in systemic blood pressure in patients with IHD as compared to healthy controls [39]. Whether this effect is due to generalized atherosclerosis causing both CAD and impaired cerebral autoregulation through damage to the cerebral microvasculature is unknown. Impaired cerebral autoregulation in IHD would fail to rapidly counteract VT-induced blood pressure changes and could, therefore, explain the observed association of ODPs with IHD. However, a possible role of cerebral autoregulation in ODPs during VT ablation requires further investigation.

### Can patients with ODPs be identified before VT ablation?

Surprisingly, measures of LVEF and acute heart failure did not predict the occurrence of ODPs. Of note, we also calculated a modified PAINESD score without NYHA classification, which did not correlate significantly with the occurrence of ODPs [40, 41]. In the case of acute heart failure, intracranial perfusion is very robust to external influences and may, therefore, not be as easily affected by water retention in acute heart failure as other organ systems. However, there was a numerical trend towards a higher prevalence of acute heart failure in patients with ODPs, so we have to consider the possibility that this study missed an association between acute heart failure and ODPs due to the limited sample size.

As LVEF was assessed at rest in sinus rhythm, it is possible that it does not reflect cardiac output during VT, when LVEF changes dramatically due to fast heart rates and aberrant conduction. Therefore, the role of cardiac output for ODPs needs further clarification. It is also noteworthy that VT cycle lengths were comparable between the two groups. However, LVEF may be a less important predictor of intracranial perfusion than local factors. These include carotid and intracerebral stenosis, microvascular ischemic disease, and cerebral emboli, which have been reported to occur in up to 58% of patients undergoing VT ablation [32].

## Study limitations

General limitations emerge from the retrospective observational and single-center study design. Recruitment of patients at an institute with expertise in VT ablation may have biased patient selection towards a more severely affected patient population with VT refractory to treatment.

Most importantly, in this study, VT induction and termination during the VT ablation procedure were not time-tracked, and continuous assessment of blood pressure values during the procedure could not be co-registered with the same device, allowing simultaneous annotation of blood pressure and ODPs. Thus, the exact temporal relationship between ODPs, blood pressure changes, and VT runs remains to be elucidated, and it cannot be excluded that other intraprocedural events such as bleeding or atrial fibrillation may have caused additional ODPs.

In addition, NIRS can be biased by changes in skin perfusion caused by vasoconstrictors, for example, or by changes in hemodynamics. It is also not a direct measure of neural function. Although it can detect cerebral hypoperfusion faster than the EEG, a critical threshold below which impairment of neurological function must be considered is still unknown. Finally, we know that neuromonitoring during surgical procedures may reduce postoperative cognitive impairment, although the evidence remains inconsistent [27, 28, 42]. However, whether ODPs during VT ablation are sufficient to induce such short- or long-term cognitive impairment remains unknown. Efforts to avoid ODPs by means of, e.g., NIRS-guided anesthesia or mechanical circulatory assist devices should only be considered if there is clear evidence of adverse clinical outcomes from ODPs [19]. The neurocognitive consequences of ODPs during VT ablation should be further investigated in prospective randomized trials.

## Conclusions

The occurrence of ventricular arrhythmias during VT catheter ablation is common and can be a hemodynamic challenge for the patient as well as a risk factor for cerebral hypoperfusion. NIRS monitoring shows that phases of cerebral oxygen desaturation occur in up to two-thirds of patients undergoing VT ablation. The occurrence of ODPs is associated with underlying IHD, a higher number of prior VT episodes, and may lead to a higher incidence of ICU admission and in-hospital VT recurrence. Long-term ablation success does not appear to be compromised. It should be carefully evaluated whether patients benefit from a routine use of neuromonitoring during VT ablation and whether prevention of ODPs and optimized management approaches may be of benefit to these patients.

## Clinical perspectives

Neuromonitoring identified phases of cerebral deoxygenation in up to two-thirds of patients undergoing VT ablation. The occurrence of cerebral deoxygenation phases was associated with an increased risk of early VT recurrence and the need for intensive care monitoring. How phases of cerebral deoxygenation are related to these observations, as well as post-procedural cognitive dysfunction, requires further clarification. To counteract cerebral hypoperfusion during VT ablation, electrical termination of the ongoing VT, the use of vasopressors, inotropes, and hemodynamic assist devices offer a broad palette of options. NIRS neuromonitoring may help to decide when to use these tools. Future studies should carefully evaluate whether patients undergoing VT ablation would benefit from the integration of NIRS neuromonitoring into the standard monitoring during VT ablation.

## Supplementary Information

Below is the link to the electronic supplementary material.

Supplementary file1 (DOCX 17853 KB)

## Data Availability

Data is available upon reasonable request from the corresponding author.

## Abbreviations

CAD: Coronary artery disease
CL: Cycle length
CRT-D: Cardiac resynchronization therapy-defibrillator
EAM: Electroanatomic mapping
ICD: Implantable cardioverter-defibrillator
ICU: Intensive care unit
IHD: Ischemic heart disease
LGE: Late gadolinium enhancement
LVEF: Left ventricular ejection fraction
MAP: Mean arterial pressure
NIRS: Near-infrared spectroscopy
ODP: Oxygen desaturation phase
PES: Programmed electrical stimulation
SD: Standard deviation
SHD: Structural heart disease
TOI: Tissue oxygenation index
VT: Ventricular tachycardia
