# Supplementary material for: A screening for cerebral deoxygenation during VT ablations in patients with structural heart disease
Source: Clin Res Cardiol. 2024 Jul 16;114(4):481–91. doi: 10.1007/s00392-024-02493-4 (PMC11946977; doi:10.1007/s00392-024-02493-4)
Supplement: Supplementary file 1 — Supplementary file1 (DOCX 17853 KB) [file 392_2024_2493_MOESM1_ESM.docx]

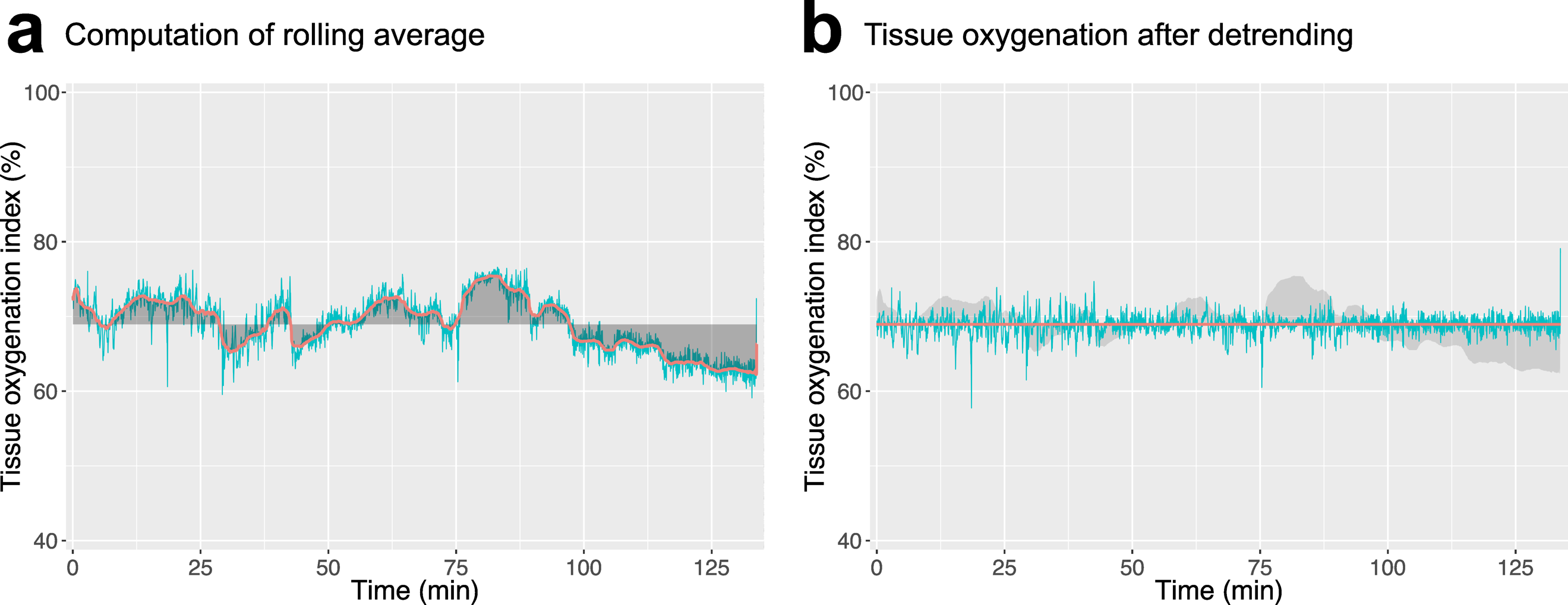


**Fig. S1** Illustration of detrending by rolling average correction using a running median with a time window of 200 sec. **(a)** Computation of running median (red line) from NIRS signal (turqouise line). Data were detrended by subtracting the difference between rolling average and average signal baseline (gray surface) from each signal point to correct for long-term NIRS signal fluctuations **(b)** TOI signal after detrending (turqouise line) with new running median corrected for long-term fluctuations in signal (red line). *NIRS = near-infrared spectroscopy;* *TOI = tissue oxygenation index*


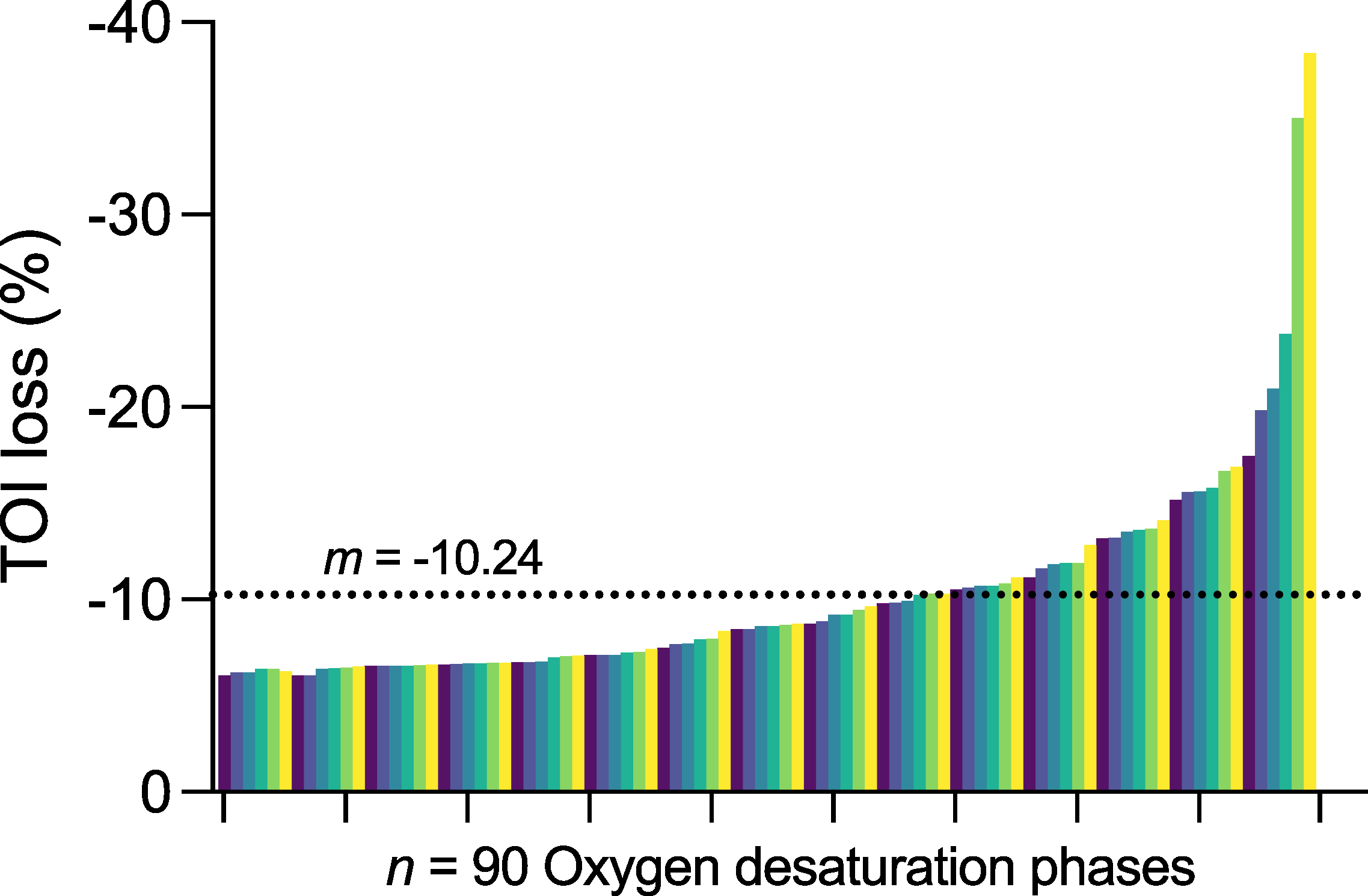


**Fig. S2** Distribution the TOI loss for each of the 90 registered oxygen desaturation phases. TOI loss is defined as the difference in TOI from baseline to the low point of a desaturation phase. *TOI = tissue oxygenation index*

**Fig. S3** Univariate linear regression of TOI loss as predicted by LVEF. Regression line plotted as solid line with dotted lines displaying 95% confidence interval. *DCM = dilated cardiomyopathy; IHD = ischemic heart disease; LVEF = left ventricular ejection fraction; SHD = structural heart disease; TOI = tissue oxygenation index*
